# Effect of plant VOCs and light intensity on growth and reproduction performance of an invasive and a native *Phytolacca* species in China

**DOI:** 10.1002/ece3.8522

**Published:** 2022-03-18

**Authors:** Danfeng Liu, Li Chen, Chao Chen, Yue Zhou, Feng Xiao, Yi Wang, Qingjun Li

**Affiliations:** ^1^ Centre for Invasion Biology, Institute of Biodiversity Yunnan University Kunming China; ^2^ Yunnan Key Laboratory of Plant Reproductive Adaptation and Evolutionary Ecology Yunnan University Kunming China

**Keywords:** invasive *Phytolacca americana*, light intensity, native *Phytolacca acinosa*, plant growth and reproduction performance, plant VOCs

## Abstract

Invasive plants often pose great threats to the growth of co‐occurring native plant species. Identifying environmental factors that facilitate exotic plant invasion and native species decline are important. In this study, we measured the effects of plant volatile organic compounds (VOCs), light intensity, and their interactions on the growth and reproduction performance of indigenous *Phytolacca acinosa*, and invasive *Phytolacca americana*, which has largely replaced the former in China. VOCs of invasive *P*. *americana* and low light levels both had negative effects on *P*. *acinosa* morphological and reproductive traits (stem length, average leaf number, total number, and length of racemes), and biomass allocation (total biomass, and leaf and flower mass fraction); low light also affected photosynthesis‐related trait (specific leaf area) of *P*. *acinosa*. In contrast, VOCs of *P*. *acinosa* had no significant effect on *P*. *americana*, but low light levels adversely affected its morphological and reproductive traits (stem length, total number, and length of racemes) and biomass allocation (total biomass, stem, and leaf mass fraction). Interactions between plant VOCs and light intensity had no significant effects on *P*. *acinosa* or *P*. *americana*. Under all experimental treatments, stem length, average leaf area, total number, and length of racemes, Root/Shoot ratio, root and flower mass fraction of *P*. *americana* were higher than those of *P*. *acinosa*, while average leaf number, specific leaf area, and leaf mass fraction was lower. These results indicated that *P*. *acinosa* was sensitive to *P*. *americana* VOCs and low light, which might affect the growth of sympatric *P*. *acinosa*. *P*. *americana* was negatively influenced by low light, but higher plant height and more reproductive organ resource allocation relative to sympatric *P*. *acinosa* might contribute to invasion success.

## Graphical abstract

*Phytolacca acinosa* was sensitive to *Phytolacca americana* VOCs and low light, which might affect the growth of sympatric *P*. *acinosa*. *P*. *americana* was negatively influenced by low light, but higher plant height and more reproductiv organ resource allocation relative to sympatric *P*. *acinosa* might contribute to invasion success.

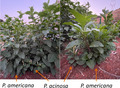

## 1 INTRODUCTION

Invasive plants often exhibit rapid growth and efficient reproduction (Assad et al., 2021; Cai et al., 2021; Luo et al., 2020; Skálová & Pyšek, 2009), and generally become dominant in the introduced range, causing serious ecological damage and economic loss (Bartz & Kowarik, 2019; Rai & Singh, 2020). Identifying environmental factors that facilitate alien plants invasion are important and helpful to understand invasion mechanisms further, and provide insights into local species protection.

The “novel weapons hypothesis” emphasizes that allelopathic compounds, for example, airborne plant volatile organic compounds (VOCs), secreted by alien plants contribute to invasion success (Barney et al., 2009; Callaway & Aschehoug, 2000; McBride et al., 2020). Plant VOCs are cues responsible for the communication among plants (Kegge & Pierik, 2010; Kigathi et al., 2019; Meents et al., 2019). The role plant VOCs play in the growth of sympatric plant species can be negative, neutral, or positive. VOCs emitted from aerial parts of invasive *Xanthium italicum* had a negative effect on the growth of surrounding plants, such as *Amaranthus mangostanus*, *Lactuca sativa*, and *Triticum aestivum* (Shao et al., 2013, 2019), while root VOCs released by *Centaurea stoebe* had a neutral to positive influence on the growth of neighbor plants (Gfeller et al., 2018). Clarifying the functions of species‐specific VOCs on plant–plant interactions is essential in understanding of the influence of invasive plants on native ones.

Compared to native plant species, invasive plants are often better competitors for light and exhibit greater adaptability to light environments. *Mikania micrantha* had been shown to grow slowly under low light, but transition to rapid growth under high light, blocking the sunlight of competing vegetation (Liu, Yan, et al., 2020; Liu, Chen, et al., 2020; Shen et al., 2015). Invasive *Rhododendron ponticum* could form larger leaf area to improve photosynthetic potential and light‐harvesting efficiency than non‐invasive *Ilex aquifolium* (Niinemets et al., 2003). *Lantana camara* adapted to moderately shaded environments by increasing leaf size, leaf biomass, and plant height (Carrión‐Tacuri et al., 2011). Moreover, light is associated with the production of plant VOCs (Gouinguené & Turlings, 2002). Seasonal change or photoperiod, which is closely related to light intensity, could affect plant volatiles emission (Effah, Barrett, Peterson, Godfrey, et al., 2020; Effah, Barrett, Peterson, Potter, et al., 2020; Hansen & Seufert, 2003; Tarvainen et al., 2005). Therefore, measurements of the interactions between plant VOCs and light intensity on invasion success are needed.

*Phytolacca americana* L., an herbaceous perennial shrub, was introduced from North America in 1935 and invaded most areas of central and southern China (Xu et al., 2012). Poisoning accidents would take place if *P*. *americana* was taken in inappropriately (Kim et al., 2005). Indigenous *Phytolacca acinosa* Roxb., a traditional Chinese medicinal plant, has been largely replaced by invasive *P*. *americana* (Xiao et al., 2019). Co‐occuring *P*. *americana* and *P*. *acinosa* showed great difference in the natural environment (Figure S1), leading to questions as to the factors that led to the decrease of *P*. *acinosa* and invasion success of *P*. *americana*. Here, plant VOCs released by aerial parts and roots of *P*. *acinosa* and *P*. *americana*, together with light intensity of 3000 lux, 2000 lux, and 1500 lux were employed to test their influence on morphological, physiological, and reproductive traits as well as resource allocation of *P*. *acinosa* and *P*. *americana*. These results would illustrate the influence of plant VOCs and light intensity on the growth and reproductive performance of native *P*. *acinosa* and invasive *P*. *americana*, and provide insights into abiotic and biotic invasion mechanisms.

## 2 MATERIALS AND METHODS

### 2.1 Plant materials

Mature racemes in the canopy of *P*. *americana* and *P*. *acinosa* were collected randomly from 15 plants in Kunming (24º49ʹ N, 102º52ʹ E) and Qujing (25º26ʹ N, 104º19ʹ E), Yunnan, China, respectively, in August, 2020. Seeds were achieved after removing the fleshly tissue of the fruits. For germinating, seeds were treated with H_2_SO_4_ to break dormancy first, and then were cultured on 1% agar medium in an artificial incubator (Yiheng, Shanghai, China). Growth conditions were set at 27ºC for 14 h light and 25ºC for 10 h dark, approximating the natural growing season of Kunming, Yunnan province (generally in April–July) except the temperature was approximate to the average temperature in the day (Liu, Yan, et al., 2020; Liu, Chen, et al., 2020). To avoid the interference of VOCs, germinating seeds of each species were cultured separately in sealed plastic boxes (13 cm × 8 cm × 6.5 cm) on 100 ml of 1% agar medium. Seeds germinated within 24 h were transferred into another set of sealed plastic boxes with soil (Jiangsu Peilei Matrix Technology Development Co., Ltd., China) for seedling establishment. After the third leaf emerged, seedlings were transplanted into pots (bottom diameter = 9 cm, top diameter = 12 cm, height = 12 cm) filled with the same soil as described above for one seedling per pot, and subjected to different treatments immediately.

### 2.2 Experimental design

We created a completely randomized design with the following factors: plant VOCs (VOCs of *P*. *acinosa* or *P*. *americana*, or no extra VOCs), light intensity (3000 lux, 2000 lux, or 1500 lux). To investigate the influence of plant VOCs on the performance of neighboring plants, VOCs produced by five plants of *P*. *acinosa* or *P*. *americana* were used separately, and no extra VOCs was set as the control. To estimate the effect of light intensity on plant performance, light intensity of 3000 lux, which is similar with the natural light environment of *P*. *acinosa* accepted in the shade of *P*. *americana* at 10 AM in the growth season, was employed as a control, and 2000 lux and 1500 lux were tested individually to represent low light environments. Plants of *P*. *acinosa* and *P*. *americana* were randomly exposed to the tested VOCs, light intensity, or their interactions, respectively. Specifically, five plants of *P*. *acinosa* grown under 3000 lux were treated as control, with each plant separated by about 8 cm. For plant VOC treatments, five seedlings of *P*. *acinosa* were co‐cultured with another five *P*. *acinosa* under 3000 lux, and another five plants of *P*. *acinosa* were also alternatively placed with five *P*. *americana* under 3000 lux. The control was five plants of *P*. *acinosa* grown without extra VOCs. For light intensity treatments, five plants of *P*. *acinosa* were grown under 2000 lux and another five *P*. *acinosa* were grown under 1500 lux. When treated by the interactions of plant VOCs and light intensity, five plants of *P*. *acinosa* were co‐cultured with another five *P*. *acinosa* under 2000 lux and 1500 lux, respectively. Meanwhile, five plants of *P*. *acinosa* were also alternatively placed with five *P*. *americana* under 2000 lux and 1500 lux, respectively. Simultaneously, same treatments described above were also conducted on *P*. *americana*. A diagram describing the experimental design was presented in Figure S2. Each treatment with five plants was repeated three times. Sufficient water was equally provided to each plant, and other environmental factors were kept the same to ensure normal growth of the plants.

In order to test the influence of VOCs on plant growth effectively, the confined space provided by artificial chambers was used. Moreover, the light intensity of the artificial chamber was precisely maintained. The space of the chamber was divided equally into three sections with the light intensity of 1500 lux, 2000 lux, and 3000 lux, respectively, which was monitored by an illumination photometer (LBJ‐20; Hangzhou Lvbo Instrument Co., Ltd, China).

### 2.3 Plant trait measurements

Total chlorophyll content of the second fully expanded leaf was determined by an UV‐330 spectrophotometer (0.3 × 0.3 cm^2^ window area; Hangzhou Lvbo Instrument Co., Ltd, China). All leaves of each plant were scanned (CanoScan LiDE 300, Canon, Japan), and their respective leaf areas were measured using ImageJ (http://rsbweb.nih.gov/ij/). Average leaf area was calculated using the total leaf area and the total number of leaves per plant. The lengths of the stem and raceme were measured with a ruler. Plants were removed from the soil and cleaned before leaves, stems, racemes, and roots of each plant were dried to a constant weight (70°C for 72 h) as measured by an analytical balance (BSA223S, OLABO, Shandong, China) with an accuracy of 0.001 g. Specific leaf area (SLA) was calculated by the total leaf area and the total leaf biomass. Root biomass and shoot biomass (leaf biomass + stem biomass + raceme biomass) were used to obtain the root/shoot (R/S) ratio (Yu et al., 2020). Resource allocation was assessed by the biomass of a particular plant tissue and the total biomass of the plant according to the formula: leaf mass fraction (LMF) = leaf biomass/total biomass of the plant × 100% (Assad et al., 2021); stem, flower, and root mass fraction (SMF, FMF, and RMF) was similarly calculated.

### 2.4 Data analysis

To test the influence of plant VOCs, light intensity, and their interactions on the morphological, physiological, and reproductive traits, together with biomass allocation of *P*. *acinosa* and *P*. *americana*, we used two‐way ANOVA (implemented in SPSS 16.0, Chicago, IL, USA) based on General Linear Models (GLMs). Before analysis, normality and homogeneity of variances were tested, and log‐transformation of the data was performed to correct deviation if necessary. Fisher's least significant difference (LSD) tests were employed to detect significant difference among treatments. Independent‐sample t‐test was also used to analyze differences between *P*. *acinosa* and *P*. *americana*.

## 3 RESULTS

According to the two‐way ANOVA analysis, plant VOCs and light intensity had different effects on plant traits of *P*. *acinosa* and *P*. *americana*, but their interactions did not (Table 1). Morphological traits, including stem length and average leaf number, reproductive traits, including total number and length of racemes, together with biomass allocation which including total biomass, LMF, and FMF of *P*. *acinosa* were affected not only by low light level but also by the VOCs of *P*. *americana*. Low light level also influenced SLA, R/S ratio, and RMF of *P*. *acinosa*, and the VOCs of *P*. *americana* also impacted its SMF. Similarly, the stem length, total number of racemes, the length of the 1st and 2nd racemes, total biomass, SMF, and LMF of *P*. *americana* were affected by low light level, but the VOCs of *P*. *acinosa* had no significant effect on *P*. *americana*. The relative content of chlorophyll, however, was not affected by the VOCs of neighbor plants or low light level.

**TABLE 1:** Two‐way ANOVA analysis of the effects of VOCs and light intensity on plant traits of *Phytolacca acinosa* and *Phytolacca americana*

Plant traits	VOCs (V)		Light intensity (L)		V × L	
	*F*	*p*	*F*	*p*	*F*	*p*
*P. acinosa*						
Stem length	**13.34**	**<.001**	**20.47**	**<.001**	1.060	.379
Average leaf number	**7.912**	.**001**	**4.899**	.**009**	0.409	.802
Average leaf area	2.741	.068	0.938	.394	1.418	.232
Specific leaf area	0.104	.902	**20.226**	**<.001**	1.289	.278
Relative content of chlorophyll	0.147	.863	0.194	.824	0.453	.770
The total number of racemes	**7.170**	.**001**	**22.57**	**<.001**	0.552	.698
Length of the 1st raceme	**7.574**	.**001**	**25.073**	**<.001**	1.600	.178
Total biomass	**21.719**	**<.001**	**23.443**	**<.001**	0.904	.464
R/S ratio	1.492	.229	**10.969**	**<.001**	0.157	.959
Root mass fraction	1.399	.251	**11.068**	**<.001**	0.236	.918
Stem mass fraction	**3.610**	.**030**	0.293	.747	0.378	.824
Leaf mass fraction	**4.795**	.**01**	**8.987**	**<.001**	0.576	.681
Flower mass fraction	**3.253**	.**042**	**21.639**	**<.001**	1.313	.269
*P. americana*						
Stem length	1.338	.266	**60.395**	**<.001**	0.888	.473
Average leaf number	0.078	.925	1.977	.143	0.137	.968
Average leaf area	0.728	.485	0.966	.383	0.223	.925
Specific leaf area	0.014	.986	0.970	.382	0.010	1.000
Relative content of chlorophyll	0.118	.889	1.514	.224	0.132	.971
The total number of racemes	0.254	.776	**4.832**	.**010**	0.375	.826
Length of the 1st raceme	1.097	.337	**14.69**	**<.001**	0.183	.947
Length of the 2nd raceme	0.072	.931	**13.62**	**<.001**	0.29	.884
Length of the 3rd raceme	0.114	.892	0.755	.472	0.059	.993
Total biomass	0.531	.589	**11.685**	**<.001**	0.088	.986
R/S ratio	0.156	.855	2.364	.098	0.005	1.000
Root mass fraction	0.422	.656	2.255	.109	0.009	1.000
Stem mass fraction	0.161	.852	**78.666**	**<.001**	1.028	.395
Leaf mass fraction	0.576	.563	**8.225**	**<.001**	0.076	.989
Flower mass fraction	0.245	.783	1.941	.148	0.225	.924

Note

*F* and *p* values were showed, with df 2, 126 for V, df 2, 126 for L, df 4, 126 for V × L. *p* values lower than .05 indicated significant differences, which were presented in bold.

**FIGURE 1:**
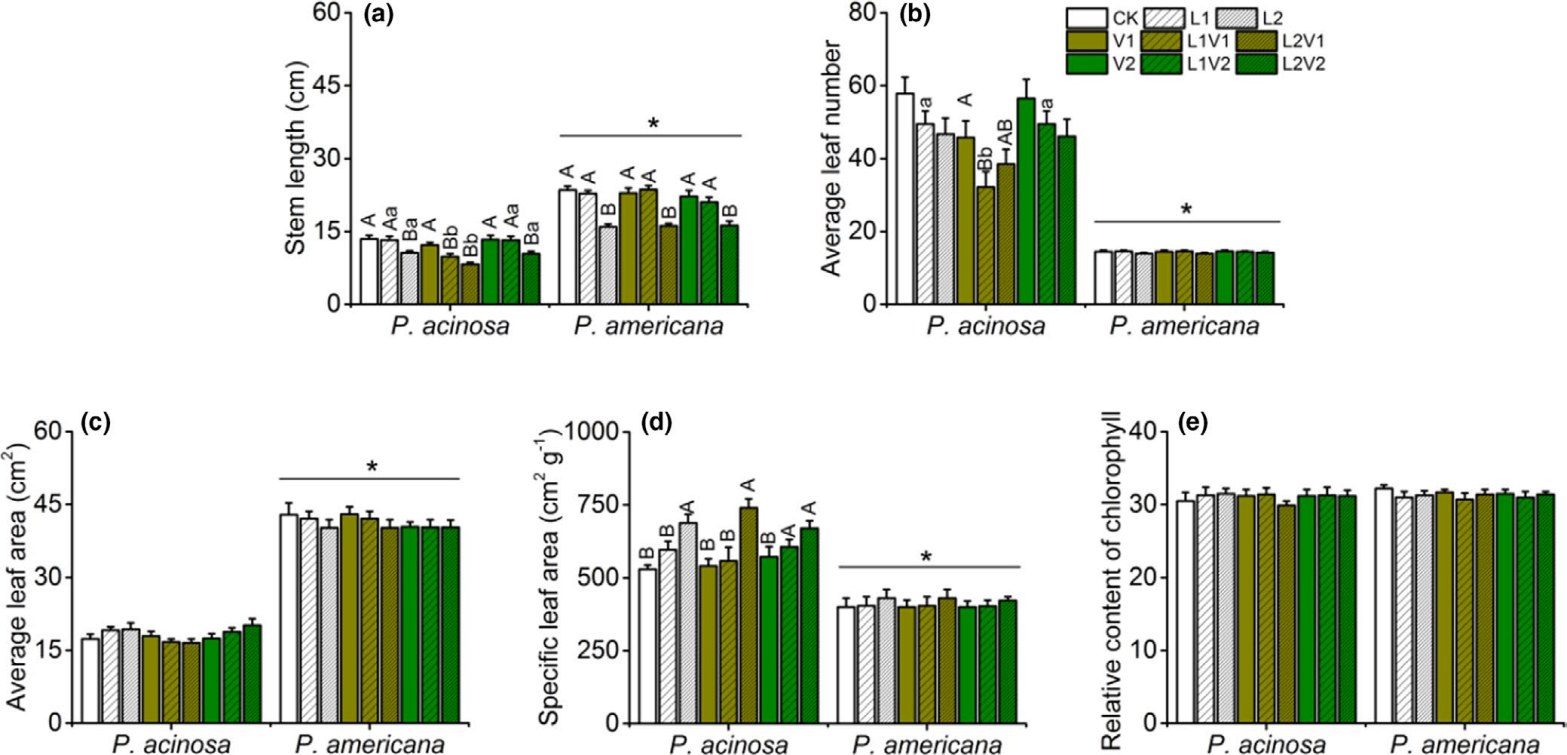
Morphological and physiological traits of native *Phytolacca acinosa* and invasive *Phytolacca americana* under the treatments of plant VOCs, light intensity, and their interactions. (a) stem length, (b) average leaf number, (c) average leaf area, (d) specific leaf area (SLA), (e) relative content of chlorophyll. CK, plants grown under 3000 lux; L1, plants grown under 2000 lux; L2, plants grown under 1500 lux; V1, plants grown with the VOCs of *P*. *americana*; L1V1, plants grown under the interaction of 2000 lux and VOCs of *P*. *americana*; L2V1, plants grown under the interaction of 1500 lux and VOCs of *P*. *americana*; V2, plants grown with the VOCs of *P*. *acinosa*; L1V1, plants grown under the interaction of 2000 lux and VOCs of *P*. *acinosa*; L2V1, plants grown under the interaction of 1500 lux and VOCs of *P*. *acinosa*. Data was shown as the means ± SE. Capital letter indicated significant difference among the treatment of light intensity, and lowercase letter suggested statistical difference among the treatment of plant VOCs, *p < .001

For plant morphological traits, stem length of *P*. *americana* was statistically longer than that of *P*. *acinosa* (Table S1). Low light resulted in short stems in both plant species, and the stem length of *P*. *acinosa* grown under low light was negatively affected by the VOCs of *P*. *americana* (Figure 1a). Average leaf number of *P*. *acinosa* was significantly higher than *P*. *americana's* (Table S1). The leaf number of *P*. *acinosa* decreased when light intensity was reduced, while the leaf number of *P*. *americana* was not significantly different under any treatment. Furthermore, leaf number of *P*. *acinosa* under low light level decreased when exposed to VOCs of *P*. *americana* (Figure 1b).

When taken photosynthesis‐related traits into consideration, there were no significant differences in average leaf areas of either species under the treatments of plant VOCs, light intensity, or their interactions, but the average leaf area of *P*. *acinosa* was statistically smaller than that of *P*. *americana* (Figure 1c; Table S1). Low light level significantly increased the SLA of *P*. *acinosa*, whether the VOCs of *P*. *acinosa* or *P*. *americana* were provided or not. However, SLA of *P*. *americana* was not significantly affected by plant VOCs or light intensity, and was smaller than *P*. *acinosa* (Figure 1d; Table S1). Relative content of chlorophyll of either species was not statistically different under any treatment (Figure 1e).

**FIGURE 2:**
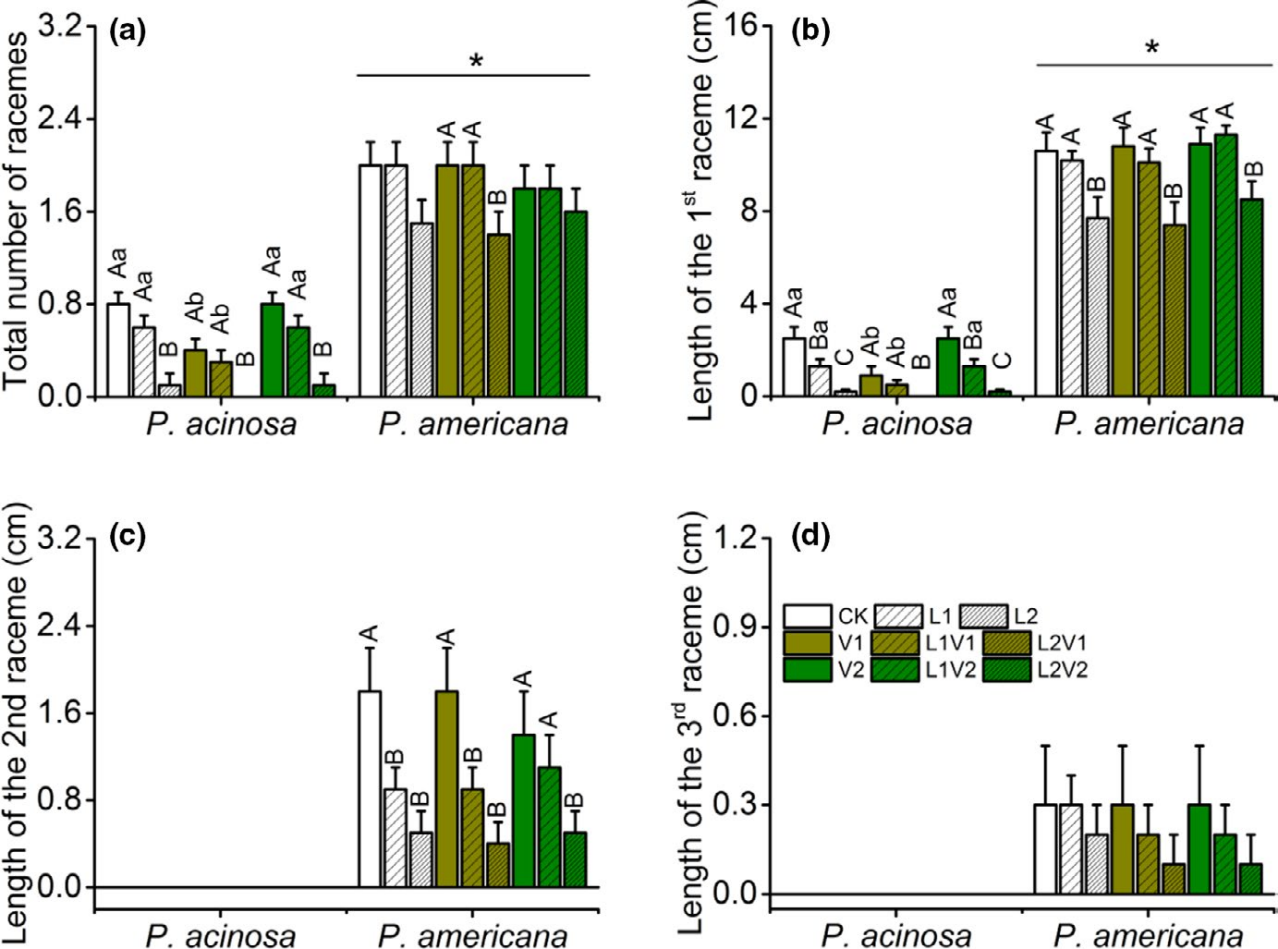
Reproductive traits of native *Phytolacca acinosa* and invasive *Phytolacca americana* under the treatments of plant VOCs, light intensity, and their interactions. (a) total number of racemes, (b) length of the 1st raceme, (c) length of the 2nd raceme, (d) length of the 3rd raceme of native *P*. *acinosa* and invasive *P*. *americana*. Treatment codes and statistical analyses were shown as in Figure 1

The reproductive traits were also showed difference among these treatments. No more than one raceme was produced by *P*. *acinosa*, while up to three were produced by *P*. *americana*. The total number of racemes of *P*. *americana* was statistically greater than *P*. *acinosa*, and low light level had a negative effect on raceme production of both species (Figure 2a; Table S1). The length of the first raceme of *P*. *americana* was significantly longer than *P*. *acinosa*, and racemes of both species shortened as light intensity decreased. The VOCs of *P*. *americana* also had a negative effect on the raceme length of *P*. *acinosa* (Figure 2b; Table S1). The second and the third raceme only found in *P*. *americana*, and the length of them both decreased as the light intensity reduced (Figure 2c‐d; Table S1).

**FIGURE 3:**
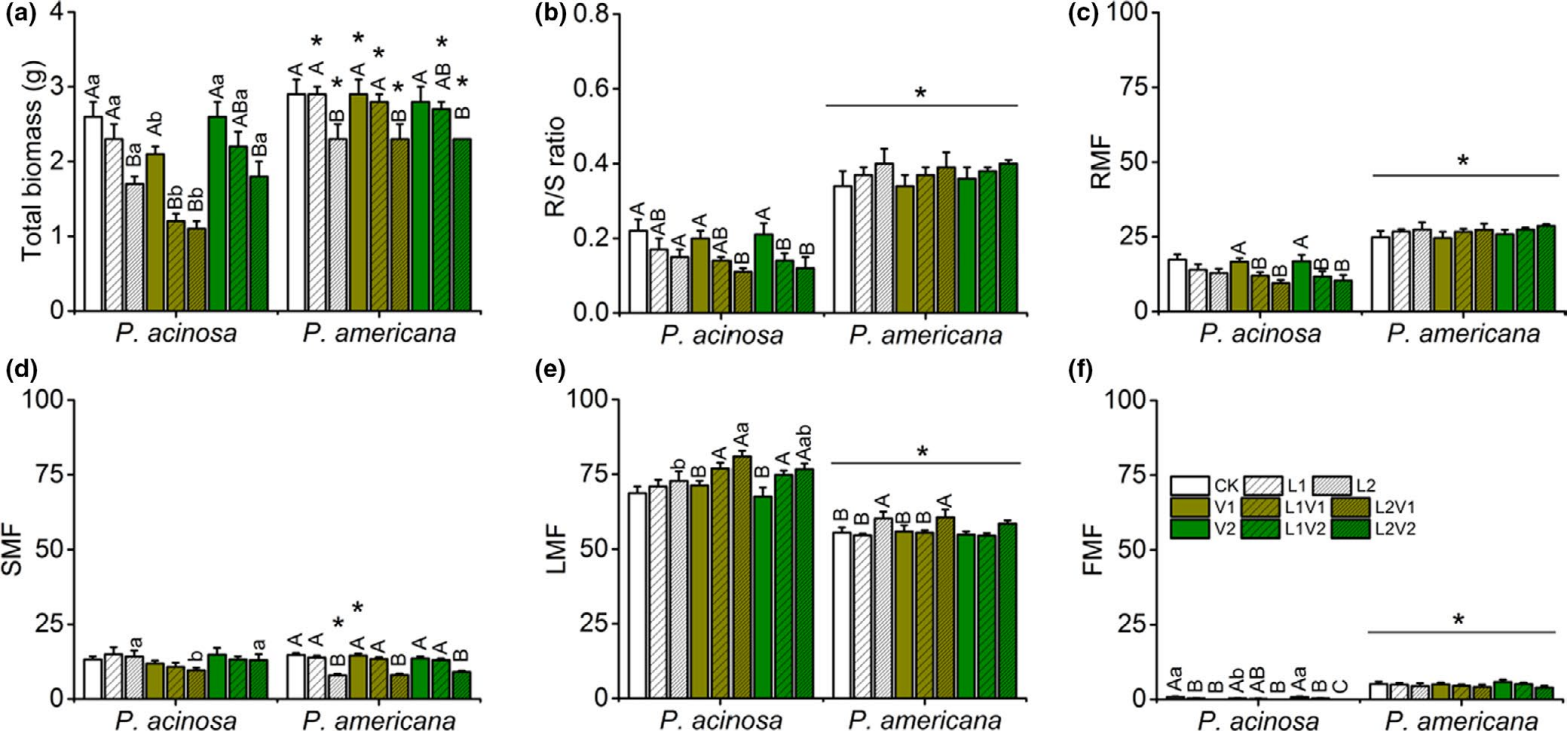
Biomass allocation of native *Phytolacca acinosa* and invasive *Phytolacca americana*, under the treatments of plant VOCs, light intensity, and their interactions. (a) total biomass, (b) root to shoot (R/S) ratio, (c) leaf mass fraction (LMF), (d) stem mass fraction (SMF), (e) root mass fraction (RMF), (f) flower mass fraction (FMF). Treatment codes and statistical analyses were shown as in Figure 1

The total biomass of both species significantly decreased with the reduced light level. The total biomass of *P*. *acinosa* was also negatively affected by the VOCs of *P*. *americana*. Except for plants grown under 3000 lux, or the combination of 3000 lux and the VOCs of *P*. *acinosa*, *P*. *acinosa* generally had lower total biomass than *P*. *americana* (Figure 3a; Table S1). The R/S ratio of *P*. *americana* was significantly higher than *P*. *acinosa*, and the R/S ratio of *P*. *americana* was slightly increased as light intensity decreased, while it significantly decreased in *P*. *acinosa* (Figure 3b; Table S1). Similarly, the RMF of *P*. *americana* was statistically higher than *P*. *acinosa*, and the RMF of *P*. *americana* increased as light intensity decreased, while it decreased greatly in *P*. *acinosa* (Figure 3c; Table S1). SMF of *P*. *americana* under 1500 lux was lower than *P*. *acinosa*, while it was higher than *P*. *acinosa* under the combination of 3000 lux and the VOCs of *P*. *americana*. Under the treatment of 1500 lux, the SMF of *P*. *acinosa* was decreased when exposed to the VOCs of *P*. *americana*. In *P*. *americana*, SMF decreased significantly under low light levels (Figure 3d; Table S1). LMF of *P*. *acinosa* was significantly higher than that in *P*. *americana*, and low light intensity increased the resource allocation to leaves of *P*. *acinosa* and *P*. *americana*. Under the treatment of 1500 lux, LMF of *P*. *acinosa* increased when exposed to the VOCs of *P*. *americana* (Figure 3e; Table S1). FMF of *P*. *americana* was significantly higher than *P*. *acinosa*, and low light level decreased FMF of both *P*. *acinosa* and *P*. *americana* (Figure 3f; Table S1).

## 4 DISCUSSION

Plant morphological and reproductive traits, together with biomass allocation, of native *P*. *acinosa* were affected by both the VOCs of invasive *P*. *americana* and light intensity. Low light level also affected photosynthesis‐related trait of *P*. *acinosa*, but for invasive *P*. *americana*, only low light level had effects on morphological and reproductive traits as well as biomass allocation. Generally, *P*. *americana* exhibited growth and reproduction advantages than *P*. *acinosa*, including longer stem, larger leaf area, greater numbers of racemes, larger total biomass, higher R/S ratio, and higher FMF, which, together, likely enhance its invasiveness, especially when in concert with its VOCs and at low light levels.

### 4.1 Invasive *P. americana* grown better than native *P. acinosa* under the effect of plant VOCs and low light level

Taller plants often capture more resources, especially light, for growth (Thomson et al., 2011), and low light level had negative impacts on the growth of *P*. *acinosa* and *P*. *americana*. VOCs of *P*. *americana* negatively affected the growth of *P*. *acinosa*, resulting in shorter stems, reduced total biomass, and LMF. About 50% aboveground biomass of *Solidago canadensis* was reduced by VOCs of invasive *Artemisia vulgaris* (Barney et al., 2009). In general, invasive species are taller than co‐occurring native species (Assad et al., 2021; Wang et al., 2018). We found that the stem length of invasive *P*. *americana* was also longer than native *P*. *acinosa* under all treatments, but the leaf number of *P*. *americana* was lower. Shorter stem length and higher leaf number suggested that native *P*. *acinosa* had higher leaf density than invasive *P*. *americana*, which might indicate that *P*. *acinosa* invested more resources in leaf construction (Feng et al., 2007). Similar results were also found that *P*. *acinosa* had higher LMF than *P*. *americana*.

High SLA is often positively correlated with high ability of resource capture, such as carbon assimilation and carbon allocation (te Beest et al., 2015; Wang et al., 2018). Invasive plants generally have larger SLA than non‐invasive plants, but similar or lower SLA of invasive species were also found (Grotkopp et al., 2010; Qin et al., 2012; van Kleunen et al., 2010; Wang et al., 2017). Native *P*. *acinosa* was more sensitive to low light level as its SLA fluctuated significantly with the changed light intensity. Even though *P*. *acinosa* and *P*. *americana* had similar content of chlorophyll, *P*. *americana* had larger leaf area and lower SLA, suggesting that *P*. *americana* consumed less resources to establish leaves, which was consistent with the lower LMF of *P*. *americana* under all the treatments.

These morphological and physiological traits were helpful for light interception of *P*. *acinosa*, while *P*. *americana* captured light by tall plant and large leaf area. The later light capture strategy might be contributed to the invasion success of *P*. *americana*, because the ability to compete for light is often treated as the primary mechanism of invasiveness (Feng et al., 2007; He et al., 2018).

### 4.2 Invasive *P. americana* showed a reproduction advantage than native *P. acinosa* when exposed to plant VOCs and low light intensities

Invasive plants tend to exhibit higher reproductive capacity than natives in order to become dominant in the community and invade habitats (Assad et al., 2021). High light level could increase the seed production of invaders (Feng et al., 2007). Invasive *P*. *americana* had more racemes than native *P*. *acinosa*, and high light level treatment increased the production of racemes of both species. The first raceme of *P*. *americana* was significantly longer than that of *P*. *acinosa*, and many *P*. *americana* produced three racemes, while *P*. *acinosa* produced no more than one. Accordingly, FMF of *P*. *americana* was higher than *P*. *acinosa*, consistent with a previous study of invasive *Rubus discolor*, which allocated more resources to the reproductive organs than non‐invasive *Rubus ursinus* (McDowell & Turner, 2002). Moreover, roots were essential for perennial plants that biomass allocation to roots made perennial weeds difficult to control (Ringselle et al., 2017). Even though invasive *P*. *americana* and native *P*. *acinosa* had similar total biomass, *P*. *americana* had higher R/S ratio and RMF than *P*. *acinosa* under all treatments. The biomass allocation to belowground or aboveground part is a trade‐off of plant to uptake water and nutrient from the soil or capture light resource, and invasive species are characterized by high resource acquisition (Feng et al., 2007; Funk, 2013; Wang et al., 2018). The biomass allocation patterns of *P*. *americana* may further enhance its invasiveness.

## 5 CONCLUSION

VOCs of the invasive *P*. *americana* adversely affected the growth performance of the native *P*. *acinosa*, as measured by stem length, average leaf number, total biomass, and LMF. Low light levels had a negative effect on the performance of both *P*. *americana* and *P*. *acinosa*, resulting in shorter stem length, lower total number and length of racemes, lower total biomass, and resource allocation. In the natural environment, *P*. *americana* are generally taller than sympatric *P*. *acinosa*, and VOCs emitted by *P*. *americana* and shade environment formed by *P*. *americana* may reduce *P*. *acinosa* growth. However, the particular volatile released by *P*. *americana* that influence the performance of *P*. *acinosa* needs to study further.

## CONFLICT OF INTEREST

The authors declare that there have no conflicts of interest.

## AUTHOR CONTRIBUTIONS

**Danfeng Liu:** Conceptualization (lead); Formal analysis (lead); Investigation (lead); Writing – original draft (lead); Writing – review & editing (lead). **Li Chen:** Formal analysis (equal); Investigation (equal). **Chao Chen:** Formal analysis (equal); Investigation (equal). **Yue Zhou:** Formal analysis (equal); Investigation (equal). **Feng Xiao:** Formal analysis (equal); Investigation (equal). **Yi Wang:** Conceptualization (supporting); Funding acquisition (supporting); Writing – review & editing (equal). **Qingjun Li:** Conceptualization (supporting); Writing – review & editing (supporting).

## Supporting information

Appendix S1

## Data Availability

All the data of the study is available at https://doi.org/10.5061/dryad.bnzs7h4cf.
