# Supplementary material for: Effect of plant VOCs and light intensity on growth and reproduction performance of an invasive and a native Phytolacca species in China
Source: Ecol Evol. 2022 Mar 18;12(3):e8522. doi: 10.1002/ece3.8522 (PMC8932221; doi:10.1002/ece3.8522)
Supplement: Supplementary file 1 — Appendix S1 [file ECE3-12-e8522-s001.docx]

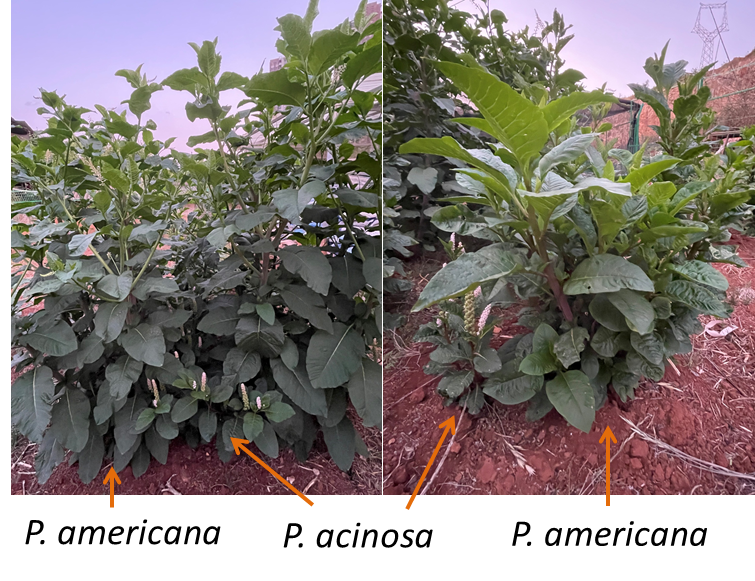


**Fig. S1 Invasive *P. americana* and native *P. acinosa* grown in the sympatric region.** *P. americana* and *P. acinosa* were grown alternatively under the natural environment in an experimental field of Laboratory of Ecology and Evolutionary Biology, State Key Laboratory for Conservation and Utilization of Bio-Resources in Yunnan, Yunnan University, and we found that *P. acinosa* was grown under the shade of *P. americana*.


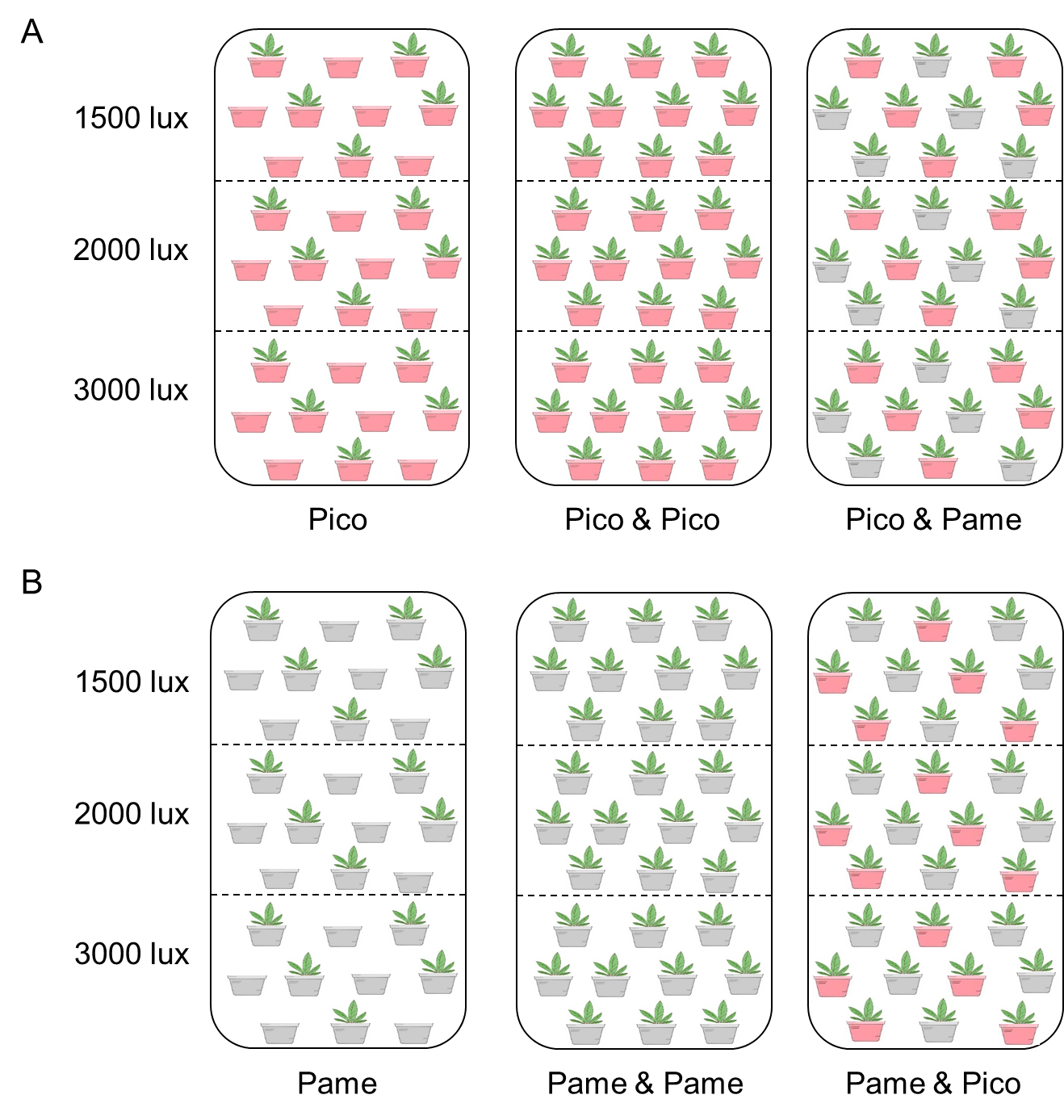


**Fig. S2 Diagram illustrating the experimental design.** A, the treatments of plant VOCs and light intensity on native *P. acinosa*; B, the treatments of plant VOCs and light intensity on invasive *P. americana*. Pico, five plants of *P. acinosa* under light intensity treatments; Pico & Pico, five plants of *P. acinosa* under the treatments of light intensity and VOCs of *P. acinosa*; Pico & Pame, five plants of *P. acinosa* under the treatments of light intensity and VOCs of *P. americana*; Pame, five plants of *P. americana* under light intensity treatments; Pame & Pame, five plants of *P. americana* under the treatments of light intensity and VOCs of *P. americana*; Pame & Pico, five plants of *P. americana* under the treatments of light intensity and VOCs of *P. acinosa*.

**Table S1 Independent-sample t-test to analyze plant traits differentce between *P. acinosa* and *P. americana* under the same treatment.**

| Plant traits | CK | | L1 | | L2 | | V1 | | L1V1 | | L2V1 | | V2 | | L1V2 | | L2V2 | |
| --- | --- | --- | --- | --- | --- | --- | --- | --- | --- | --- | --- | --- | --- | --- | --- | --- | --- | --- |
|  | t | *P* | t | *P* | t | *P* | t | *P* | t | *P* | t | *P* | t | *P* | t | *P* | t | *P* |
| stem length | -8.502 | <0.001 | -9.719 | <0.001 | -7.083 | <0.001 | -9.868 | <0.001 | -13.780 | <0.001 | -12.299 | <0.001 | -6.195 | <0.001 | -6.253 | <0.001 | -5.790 | <0.001 |
| average leaf number | 9.580 | <0.001 | 9.669 | <0.001 | 7.423 | <0.001 | 6.913 | <0.001 | 4.043 | 0.001 | 6.157 | <0.001 | 7.993 | <0.001 | 9.735 | <0.001 | 6.748 | <0.001 |
| average leaf area | -9.755 | <0.001 | -13.495 | <0.001 | -9.771 | <0.001 | -13.847 | <0.001 | -15.359 | <0.001 | -12.848 | <0.001 | -16.140 | <0.001 | -12.298 | <0.001 | -10.066 | <0.001 |
| specific leaf area | 3.843 | 0.001 | 4.524 | <0.001 | 6.145 | <0.001 | 3.992 | <0.001 | 2.715 | 0.012 | 7.165 | <0.001 | 4.224 | <0.001 | 6.056 | <0.001 | 8.486 | <0.001 |
| relative content of chlorophyll | -1.288 | 0.214 | 0.215 | 0.831 | 0.228 | 0.822 | -0.474 | 0.641 | 0.616 | 0.543 | -1.652 | 0.110 | -0.247 | 0.807 | 0.222 | 0.826 | -0.142 | 0.888 |
| the total number of racemes | -6.000 | <0.001 | -6.548 | <0.001 | -5.991 | <0.001 | -7.483 | <0.001 | -8.404 | <0.001 | -6.548 | <0.001 | -4.410 | <0.001 | -5.020 | <0.001 | -6.313 | <0.001 |
| length of the 1st raceme | -8.930 | <0.001 | -15.952 | <0.001 | -8.340 | <0.001 | -11.407 | <0.001 | -16.116 | <0.001 | -7.712 | <0.001 | -9.619 | <0.001 | -19.007 | <0.001 | -9.863 | <0.001 |
| length of the 2nd raceme | -4.559 | <0.001 | -5.208 | <0.001 | -2.281 | 0.039 | -4.559 | <0.001 | -5.208 | <0.001 | -2.394 | 0.031 | -3.688 | 0.002 | -4.199 | 0.001 | -2.503 | 0.025 |
| length of the 3rd raceme | -1.643 | 0.123 | -1.742 | 0.103 | -1.787 | 0.096 | -1.653 | 0.120 | -1.757 | 0.101 | -1.442 | 0.171 | -1.747 | 0.103 | -1.792 | 0.095 | -1.799 | 0.094 |
| total biomass | -1.250 | 0.222 | -2.868 | 0.008 | -2.703 | 0.012 | -3.954 | <0.001 | -10.197 | <0.001 | -5.894 | <0.001 | -0.779 | 0.442 | -2.499 | 0.021 | -2.903 | 0.010 |
| R/S ratio | -2.737 | 0.011 | -6.387 | <0.001 | -4.873 | <0.001 | -3.275 | 0.003 | -9.904 | <0.001 | -6.056 | <0.001 | -3.570 | 0.001 | -9.263 | <0.001 | -10.165 | <0.001 |
| root mass fraction | -2.672 | 0.012 | -6.329 | <0.001 | -5.507 | <0.001 | -3.466 | 0.002 | -9.851 | <0.001 | -7.431 | <0.001 | -3.538 | 0.001 | -8.257 | <0.001 | -9.143 | <0.001 |
| stem mass fraction | -1.092 | 0.284 | 0.449 | 0.657 | 2.962 | 0.009 | -2.188 | 0.037 | -1.761 | 0.089 | 1.542 | 0.134 | 0.509 | 0.618 | 0.110 | 0.913 | 1.949 | 0.070 |
| leaf mass fraction | 4.813 | <0.001 | 6.705 | <0.001 | 3.205 | 0.003 | 5.935 | <0.001 | 10.155 | <0.001 | 6.302 | <0.001 | 3.981 | 0.001 | 11.933 | <0.001 | 8.372 | <0.001 |
| flower mass fraction | -4.997 | <0.001 | -7.830 | <0.001 | -4.351 | <0.001 | -7.130 | <0.001 | -8.689 | <0.001 | -4.782 | <0.001 | -6.177 | <0.001 | -9.060 | <0.001 | -6.350 | <0.001 |

CK, plants grown under 3000 lux; L1, plants grown under 2000 lux; L2, plants grown under 1500 lux; V1, plants grown with the VOCs of *P. americana*; L1V1, plants grown under the interaction of 2000 lux and VOCs of *P. americana*; L2V1, plants grown under the interaction of 1500 lux and VOCs of *P. americana*; V2, plants grown with the VOCs of *P. acinosa*; L1V1, plants grown under the interaction of 2000 lux and VOCs of *P. acinosa*; L2V1, plants grown under the interaction of 1500 lux and VOCs of *P. acinosa*.
